# Regional variation in climate change alters the range‐wide distribution of colour polymorphism in a wild bird

**DOI:** 10.1002/ece3.10311

**Published:** 2023-07-17

**Authors:** Katja Koskenpato, Aleksi Lehikoinen, Chiara Morosinotto, Ruslan Gunko, Patrik Karell

**Affiliations:** ^1^ Bioeconomy Research Team Novia University of Applied Sciences Ekenäs Finland; ^2^ Finnish Museum of Natural History, The Helsinki Lab of Ornithology University of Helsinki Helsinki Finland; ^3^ Department of Biology Lund University Lund Sweden; ^4^ Department of Biology University of Turku Turku Finland; ^5^ Present address: Department of Forest Sciences, Faculty of Agriculture and Forestry University of Helsinki Helsinki Finland; ^6^ Present address: Department of Biology University of Padova Padova Italy; ^7^ Present address: National Biodiversity Future Center (NBFC) Palermo Italy; ^8^ Present address: Department of Ecology and Genetics University of Uppsala Uppsala Sweden

**Keywords:** biogeography, climate change, colour polymorphism, Gloger's rule, melanism, *Strix aluco*

## Abstract

According to Gloger's rule, animal colouration is expected to be darker in wetter and warmer climates. Such environmental clines are predicted to occur in colour polymorphic species and to be shaped by selection if colour morphs represent adaptations to different environments. We studied if the distribution of the colour polymorphic tawny owl (*Strix aluco*) morphs (a pheomelanic brown and a pale grey) across Europe follow the predictions of Gloger's rule and if there is a temporal change in the geographical patterns corresponding to regional variations in climate change. We used data on tawny owl museum skin specimen collections. First, we investigated long‐term spatiotemporal variation in the probability of observing the colour morphs in different climate zones. Second, we studied if the probability of observing the colour morphs was associated with general climatic conditions. Third, we studied if weather fluctuations prior to the finding year of an owl explain colour morph in each climate zone. The brown tawny owl morph was historically more common than the grey morph in every studied climate zone. Over time, the brown morph has become rarer in the temperate and Mediterranean zone, whereas it has first become rarer but then again more common in the boreal zone. Based on general climatic conditions, winter and summer temperatures were positively and negatively associated with the proportion of brown morph, respectively. Winter precipitation was negatively associated with the proportion of brown morph. The effects of 5‐year means of weather on the probability to observe a brown morph differed between climate zones, indicating region‐dependent effect of climate change and weather on tawny owl colouration. To conclude, tawny owl colouration does not explicitly follow Gloger's rule, implying a time and space‐dependent complex system shaped by many factors. We provide novel insights into how the geographic distribution of pheomelanin‐based colour polymorphism is changing.

## Graphical abstract

We use data from 1900 to 2016 on tawny owl museum specimens hosted in different museums and institutes across Europe. We show that the probability that observed tawny owl individuals are pheomelanic brown is largely explained by the climate zone in which they are observed as well as by climatic variables. Our results indicate that climate change plays a great role in shaping the distribution patterns of tawny owl colour morphs (a pheomelanic brown and a pale grey) in Europe.

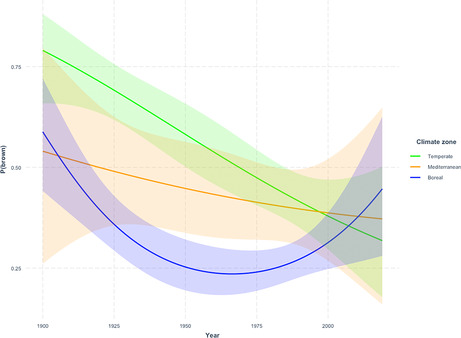

## 1 INTRODUCTION

In brief, Gloger's rule states that homeothermic animals are lighter coloured the further away from the equator they live and predicts darker‐coloured individuals in warm and humid regions (Delhey, 2019; Rensch, 1929). Although general patterns supporting the rule have been widely reported (Amar et al., 2014; Delhey et al., 2019; Marcondes et al., 2021; Passarotto et al., 2021; Romano et al., 2018; Roulin et al., 2011), the generality of the rule and why it occurs are still poorly understood (Delhey, 2019). This is because the proximate mechanisms for why Gloger's rule occurs remain unresolved, although most of the literature suggests that animal colouration is somehow linked to climatic variables, such as humidity and temperature (Delhey, 2019). Eumelanins and pheomelanins are the specific pigments in vertebrate integument expected to vary in intensity according to Gloger's rule. However, two versions have been proposed to explain how these pigments can vary according to climate. First, the simple version of Gloger's rule states that both eumelanin and pheomelanin deposition increases with temperature and humidity (Delhey, 2017, 2019). In contrast, the second and more complex version predicts that eumelanin deposition increases with humidity and temperature, whereas pheomelanin increases only with temperature but decreases with humidity (Delhey, 2017, 2019). An understanding of the underlying mechanisms could, however, clarify which pattern to expect. Several theories of why Gloger's rule occurs have been proposed, dealing mainly with camouflage, photoprotection, immunity against parasites and pleiotropic effects between pigmentation and physiological functions (Delhey, 2017; Roulin, 2014). As lower latitudes usually host more vegetation and less snow, thus making the landscape dark, it is thought that dark integument is of better camouflage value in these areas. Also, dark colouration may be beneficial at lower latitudes against high ultraviolet radiation and greater parasite loads (via, e.g. photoprotection, melanin‐induced wear resistance and pleiotropic effects regarding immunity and metabolism (Delhey, 2017; Koskenpato et al., 2020; Roulin, 2014)).

In colour polymorphic species, the individuals display genetically different colour morphs (Buckley, 1987; Ford, 1945; Roulin, 2004) that are predicted to have different sensitivity to the environment (Roulin, 2004). Thus, environmental changes will affect the different colour morphs in different ways, making melanin‐based colour polymorphic species ideal objects when studying the predictions of Gloger's rule and the environmental factors driving them. There is already some evidence that colour‐polymorphic bird species follow the predictions of Gloger's rule (Amar et al., 2014). However, to study large‐scale geographical and temporal phenomena in relation to climate change, we need long‐term studies, which to date are scarce (but see Delhey et al., 2020; Romano et al., 2018; Tian & Benton, 2020). Also, the changes in animal colouration frequencies may be region‐specific, as different climate zones and latitudes face climate change in different manners, for example in terms of precipitation and temperature changes (Delhey et al., 2020). Therefore, we would predict changes in the distribution of melanin‐based colouration both within and between species according to the predictions of Gloger's rule (Delhey, 2019). Climate change is also expected to lead to regional increases in vegetation, desertification, UV radiation and parasite loads, which are the factors predicted to underlie colouration‐specific adaptations that constitute Gloger's rule (Roulin, 2014). In Europe, climate warming has been strongest in the Iberian Peninsula, central‐ and north‐eastern Europe during summers since the 1960s, whereas Northern Europe has faced the strongest warming during winters (EEA, 2017). At the same time, annual precipitation has increased in north‐eastern and north‐western Europe and decreased in parts of southern Europe. Mean summer precipitation has significantly decreased in most of southern Europe, whereas a significant increase has been observed in some parts of northern Europe, especially during winter (EEA, 2017). We would therefore expect that animal colouration would change accordingly in different regions of the continent.

In this paper, we use the tawny owl (*Strix aluco*) as a study species to investigate how climate change may have affected Gloger's rule gradients in Europe. The tawny owl is a colour polymorphic species displaying two genetically different morphs: a pheomelanistic reddish‐brown (hereafter brown) and a pale grey (hereafter grey) morph (Brommer et al., 2005; Gasparini et al., 2009; Karell et al., 2011). Both eumelanins and pheomelanins are involved in shaping the tawny owl colouration, but most of the between‐morph variation in colouration is due to pheomelanins (Gasparini et al., 2009). Tawny owls are distributed across the Western Palearctic, with both colour morphs occurring across the range, but their relative abundances vary regionally (Galeotti & Cesaris, 1996). Based on samples collected from part of tawny owls' breeding range in West, Central and South Europe, it has been proposed that the brown morph prefers warm and humid environments, whereas the grey morph prefers cool and dry environments (Galeotti & Cesaris, 1996) and thus the colouration of this species seems to follow Gloger's rule. Similar patterns of latitudinal variation in colouration have been detected in the colour‐polymorphic screech owl (*Megascops asio*; Gehlbach, 1994), which displays a similar bimodal pheomelanin‐based colour variation as the tawny owl.

In Northern Europe, where tawny owls live in their northernmost range margin, the brown morph suffers higher mortality in cold and snow‐rich winters compared to the grey morph. However, this survival difference between morphs is absent in winters with less snow (Karell et al., 2011). In Northern Europe, winters have become milder since the 1960s and accordingly, the survival selection against the brown morph has disappeared and the frequency of the brown morph has rapidly increased (Karell et al., 2011). The survival difference between the colour morphs under snowy conditions could be due to better plumage insulation capacity (Koskenpato et al., 2016) or better camouflage of the grey morph in snowy northern landscapes compared to the brown morph (Koskenpato et al., 2020). On the other hand, the brown morph seems to have higher fitness in lower latitudes with presumably more productive environments. In a central European population, the brown morph was found to have a higher survival rate than the grey morph and brown males have a rather constant reproductive effort regardless of environmental factors, while the grey morphs are more flexible (Emaresi et al., 2014). Furthermore, brown pairs produce heavier fledglings than grey ones (Morosinotto et al., 2020) and brown offspring convert food to growth more efficiently than grey ones (Piault et al., 2009). These life history differences between morphs may be linked with behavioural and ecological traits. Indeed, the brown morph uses a wider diversity of prey than the grey morph and can therefore be viewed as more of a generalist predator than the grey morph, which further indicates that the brown morph may be better at utilising more productive environments (Karell et al., 2021). Also, there is some evidence of differential habitat selection of morphs, as the brown morph prefers denser woods (Galeotti & Sacchi, 2003). These morph‐specific differences suggest that we can expect large‐scale spatial variation in the presence of the colour morphs which is modified by climate change.

Here, we conduct a large‐scale spatiotemporal survey of the presence of tawny owl colour morphs across Europe. The aim is to analyse if tawny owl colouration follows the simple version of Gloger's rule (i.e. plumage darkness increases with humidity and temperature) throughout its distribution, and if there are long‐term temporal changes in these patterns. We base our predictions on the simple version of Gloger's rule because previous studies of owls support this pattern rather than the complex one (Galeotti et al., 2009; Galeotti & Cesaris, 1996; Gehlbach, 1994; Passarotto et al., 2021). The alternative to Gloger's rule, the thermal melanism hypothesis (also known as Bogert's rule), predicts that animals are darker in colder environments, as a darker integument heats faster than a paler one and gives advance in reaching thermal equilibrium. The thermal melanism hypothesis mainly applies to ectotherms (Clusella‐Trullas et al., 2007; Farquhar et al., 2022; Hantak et al., 2022), but recent studies have suggested that it could also apply to endothermic birds (Amar et al., 2019; Delhey et al., 2019; Galván et al., 2018; Romano et al., 2018).

First, we study long‐term temporal changes in the colour morph frequencies within climate zones to assess climate zone‐specific patterns in the occurrence of the morphs. We use one of the most widely used climate classification systems, the Köppen‐Geiger classification (Rubel & Kottek, 2010), which is based on seasonal precipitation and temperature patterns. Second, we study if general climatic conditions (long‐term weather means) explain colour morph occurrence regardless of climate zones. Third, we study weather as 5‐year means prior to the observation of an owl explain which colour morph is more likely to be found in each climate zone. We hypothesise that the brown morph is more dominant in humid oceanic climates (western temperate and Mediterranean zones), whereas the grey morph is dominant in colder and drier continental parts of Northern and Eastern Europe (boreal zone). We further hypothesise that climate zone‐specific colouration patterns show regional responses to climate change in the same manner. According to the simple version of Gloger's rule, we expect that high temperature and precipitation are associated with a higher proportion of brown morph.

## 2 MATERIALS AND METHODS

### 2.1 Data collection

We collected information on 1053 adult tawny owl specimens from 1900 to 2016 hosted by 19 different European museums and institutes (listed in Data S1). We either colour‐scored the specimens right at the museum or took pictures from the chest, back and side of the owls and colour‐scored them afterwards based on the pictures. The colour scoring method we used is based on the amount of reddish‐brown in the facial disc, chest, back and overall appearance of the owls (Brommer et al., 2005; Karell et al., 2013), and results in a bimodally distributed score ranging between 4 and 14 points, where the grey morph has scored 4–9 and the brown morph has scored 10–14 (see Brommer et al., 2005 for details on the scoring). We believe that the potential fading of reddish pigments of plumage over time in older museum specimens (Armenta et al., 2008) does not pose a problem here, as we only categorise the specimens as grey or brown. Even a slightly faded reddish pigment will give the impression of a brown, rather than a grey, individual. We documented the finding year and location of each owl as exactly as possible and converted the locations to correspond to the WGS84 coordinate system. If coordinates were not provided by the museum, we extrapolated the location in Google Maps by using the most accurate location information provided, for example city or region centre. In most cases, the cause of death of an owl was not provided for the museum specimens and could not be taken into account. Some of the old specimens were collected by shooting the owl, but mainly the causes of death (if provided) were accidents, starvation or injuries. We assume that the museum specimens represent a sample of the population living in a particular area in a particular year (i.e. the likelihood of finding a specific colour morph in a given location is because it is more abundant in that location).

### 2.2 Repeatability of colour scoring

The colour scoring of the tawny owl specimens was done by KK, CM and PK. To ensure that the colour scoring is repeatable we randomly selected 33 pictures of museum specimens and tested the colour scoring repeatability across the three observers. The repeatability for facial disc percentage (0%–100% of brown colouration) was *r* = 0.92 ± 0.025 SE (CI 0.858, 0.953; *p* < .001), whereas for the overall colour morph (grey or brown) was *r* = 0.864 ± 0.097 SE (CI 0.623, 0.987; *p* < .001). Repeatability was calculated using the ‘rptR’ package in R (Stoffel et al., 2017).

### 2.3 Environmental variables

**FIGURE 1:**
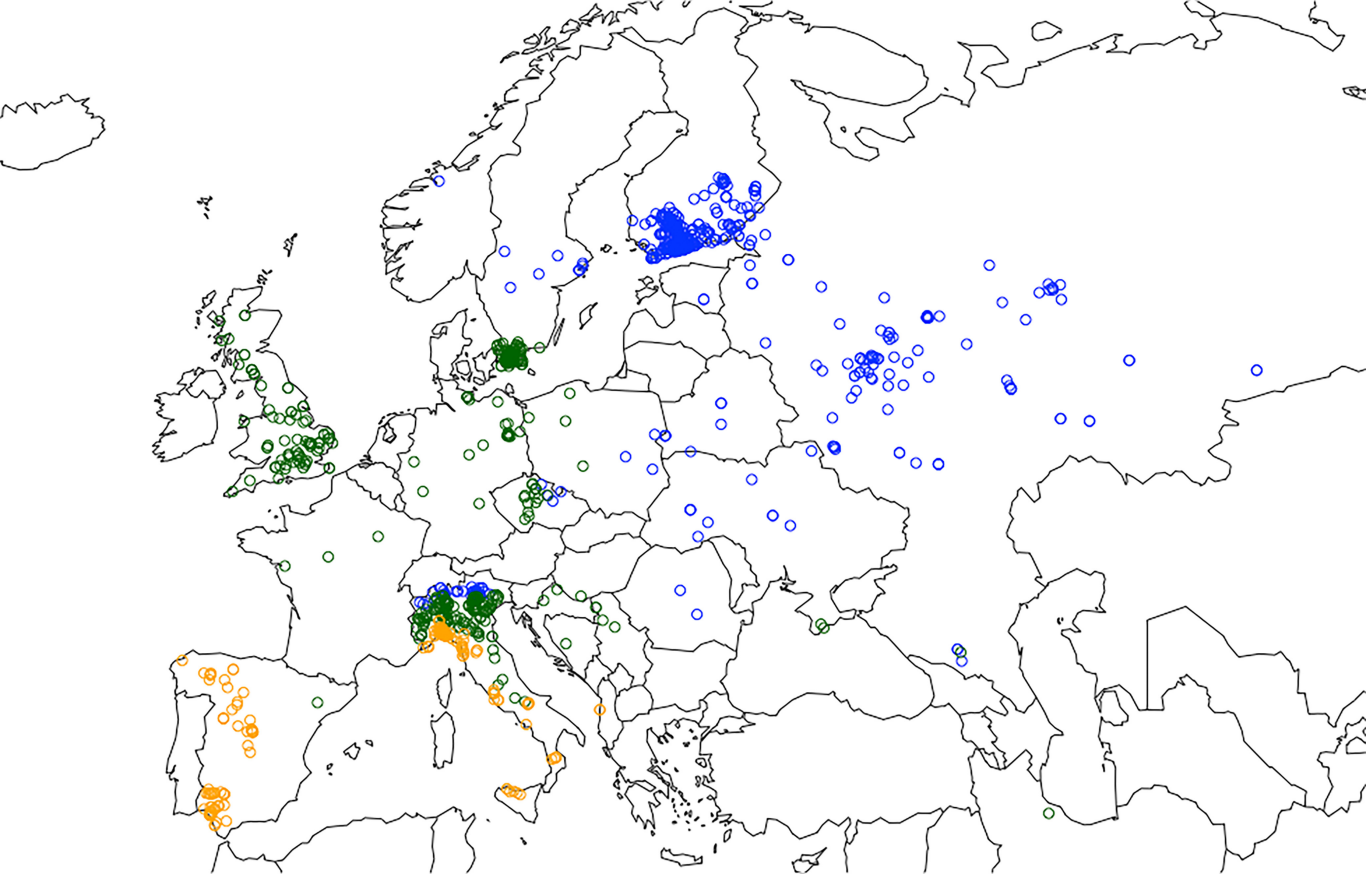
Map showing the finding location of owl specimens (1900–2016) used in this study. Blue dots are data for boreal zone, green dots for temperate zone and yellow dots for the Mediterranean zone.

We wanted to test if a proxy for the frequency of colour morphs, the probability of observing a brown colour morph, ‘*p*(brown)’, is dependent on climate zone and year. We divided the owl specimens according to their finding locations into three climate zones within the Köppen‐Geiger climate classification (Rubel & Kottek, 2010) considering data from 1951 to 1975, that is the mid‐study period of our dataset (older and newer classifications differed very little from it; data downloaded from http://koeppen‐geiger.vu‐wien.ac.at/shifts.htm). Overall, we considered 511 owls within the boreal zone (including climate groups Dfa, Dfb, Dfc and E), 381 owls within the temperate zone (including climate groups Cfa, Cfb and Cfc) and 161 owls within the Mediterranean zone (including climate groups Csa and Csb, Figure 1). We hereafter refer to this data set from 1900 to 2016 as “historical data”.

We obtained annual winter temperature and winter precipitation (December–February), and summer temperature and summer precipitation (June–August) starting from 1950 (from the oldest data available) from the 0.25‐degree regular grid dataset provided by European Climate Assessment & Dataset (www.ecad.eu/download/ensembles/download.php). As climate zones (described above) do not test explicitly the effect of weather, we used long‐term means (1950–2016) of these weather variables to explain *p*(brown) in the historical data regardless of climate zones. In addition, we test if short‐term means of weather as 5 years prior to observation of an owl would explain the probability of brown morph in each climate zone. We hereafter refer to this as a “5‐year mean” of temperature or precipitation. By using this approach, we estimate and test the effects of the weather conditions the owl has experienced on the probability that we observe it. As the weather data starts only from 1950, the 5‐year means of weather variables were calculated for owl specimens found in 1955 and after. This resulted in sample sizes of 337 for the boreal zone, 227 for the temperate zone and 114 for the Mediterranean zone. We hereafter refer to these data from 1955 to 2016 as “weather data”.

### 2.4 Statistical analyses

#### 2.4.1 Climate zone model (historical data 1900–2016)

We used a general linear model (GLM) with binomial error distribution to explain the probability of an owl being brown (‘*p*(brown)’, binomial factor: 0/1) depending on climate zone (categorical factor: boreal/temperate/Mediterranean) and year when the owl specimen was discovered (continuous variable). ‘Year’ and ‘Year^2^’ were entered as continuous fixed effects to be able to assess temporal trends conditional on the climate zone. The conducted prediction‐based model was: *colour morph* ~ year*climate zone + *year* ^2^*climate zone, where the temperate zone was set as a reference category.

#### 2.4.2 General climatic conditions model (historical data 1900–2016)

We used a generalised linear mixed model (GLMER) with binomial error distribution to analyse if *p*(brown), binomial factor: 0/1, is explained by long‐term (1950–2016) means of summer temperature, summer precipitation, winter temperature or winter precipitation. Year when the owl specimen was found was entered as random effect. Correlations between weather variables were tested, and in all cases met the criterion of |*r*| < 0.7 (Dormann et al., 2013). All weather variables in the model were standardised to mean values ±1 SD to enable the comparison of coefficients. The conducted prediction‐based model was: *colour morph* ~ *1*|*year* + *winter temperature* + *winter precipitation* + *summer temperature* + *summer precipitation*. The model was conducted using the ‘lme4’ package in R (Bates et al., 2015). We also conducted the general climatic conditions model separately for each climate zone (see Data S1).

#### 2.4.3 Short‐term weather models (weather data 1955–2016)

We used a GLM with binomial error distribution to explain the probability of an owl being brown (‘*p*(brown)’, binomial factor: 0/1) depending on 5‐year means of summer/winter precipitation and temperature prior to the observation of the owl in the locality it was observed in. Models were conducted separately for each climate zone. Weather variables were standardised to mean values ±1 SD to enable a comparison of coefficients. Correlations between weather variables were tested, and the correlation was high (|*r*| > 0.7, Dormann et al., 2013) between summer temperature and winter temperature, and winter temperature and summer precipitation in the Mediterranean zone. Thus, separate models for winter and summer weather were conducted for the Mediterranean zone. Our conducted prediction‐based models were:

Boreal zone: *colour morph* ~ *5*‐*year mean winter temperature* + *5*‐*year mean winter precipitation* + *5*‐*year mean summer temperature* + *5*‐*year mean summer precipitation*.

Temperate zone: *colour morph* ~ *5*‐*year mean winter temperature* + *5*‐*year mean winter precipitation* + *5*‐*year mean summer temperature* + *5*‐*year mean summer precipitation*.

Mediterranean zone winter: *colour morph* ~ *5*‐*year mean winter temperature* + *5*‐*year mean winter precipitation*.

Mediterranean zone summer: *colour morph* ~ *5*‐*year mean summer temperature* + *5‐year mean summer precipitation*.

We conducted respective models for 1‐ and 3‐year means to detect whether there were differences when considering whether variables closer to the finding of the specimens and compared all three model types using the Akaike information criterion, AIC, (Burnham & Anderson, 2002; see Data S1). In all conducted models, *p* < .05 was considered to be statistically significant.

Residuals of all applied models were inspected for spatial autocorrelation by using the ‘ncf’ package in R (Bjornstad, 2022) and no significant spatial autocorrelation was found (see correlograms in Figures S1–S14).

All the statistics were conducted in R version 4.2.0 (R Core Team, 2021).

## 3 RESULTS

### 3.1 Climate zone‐specific long‐term trends in *p*(brown), 1900–2016

**FIGURE 2:**
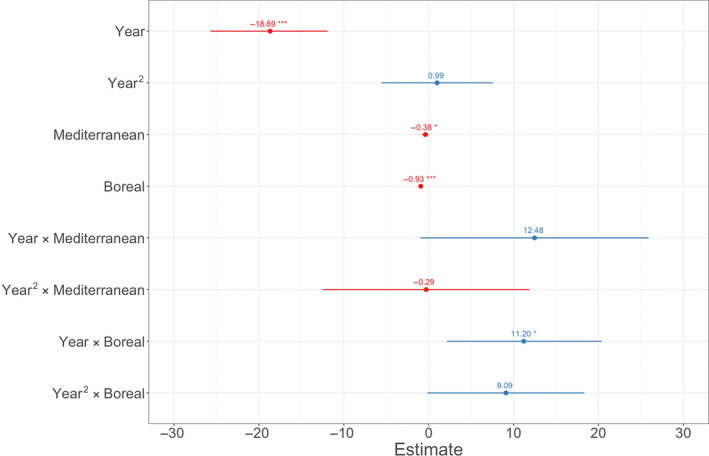
Variable estimates of GLM explaining the temporal patterns in the probability of an owl being brown (1900–2016) with climate zones. Temperate zone is the reference category, Year^2^ = polynomic year, asterisks indicate the significance level of the *p*‐values (*** = <.001, ** = <.01, * = <.05).

**FIGURE 3:**
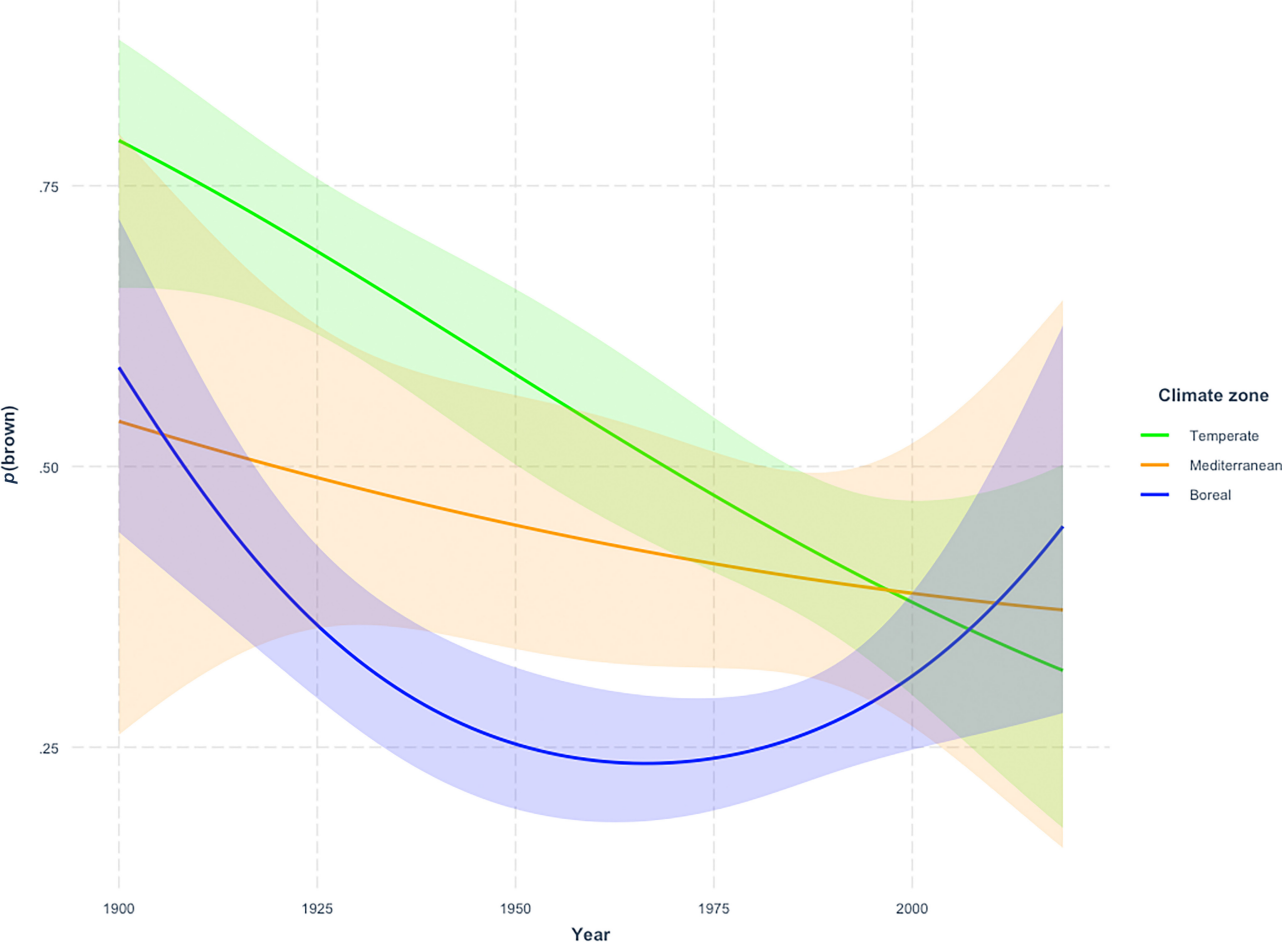
Predicted probabilities (estimates ± CI of the GLM) for *p*(brown) in each climate zone in historical data (1900–2016) according to the climate zone model (Table S1).

The boreal‐ and Mediterranean zones had a significantly lower probability of brown owls than the temperate zone (Figure 2). The trends over time varied between climate zones (Figure 3): *p*(brown) significantly decreased linearly over time in the temperate zone and in the Mediterranean zone (Figures 2 and 3). There was however a non‐significant tendency that *p*(brown) decreased less over time in Mediterranean zone compared to the temperate zone (Figure 3). Contrary to the other climate zones *p*(brown) showed a positive linear trend and a tendency for a quadratic trend over time in the boreal zone, where *p*(brown) first decreased but then increased in recent decades (Figures 2 and 3). Full model output is shown in Table S1.

### 3.2 Associations between *p*(brown) and general climatic conditions, 1900–2016

**FIGURE 4:**
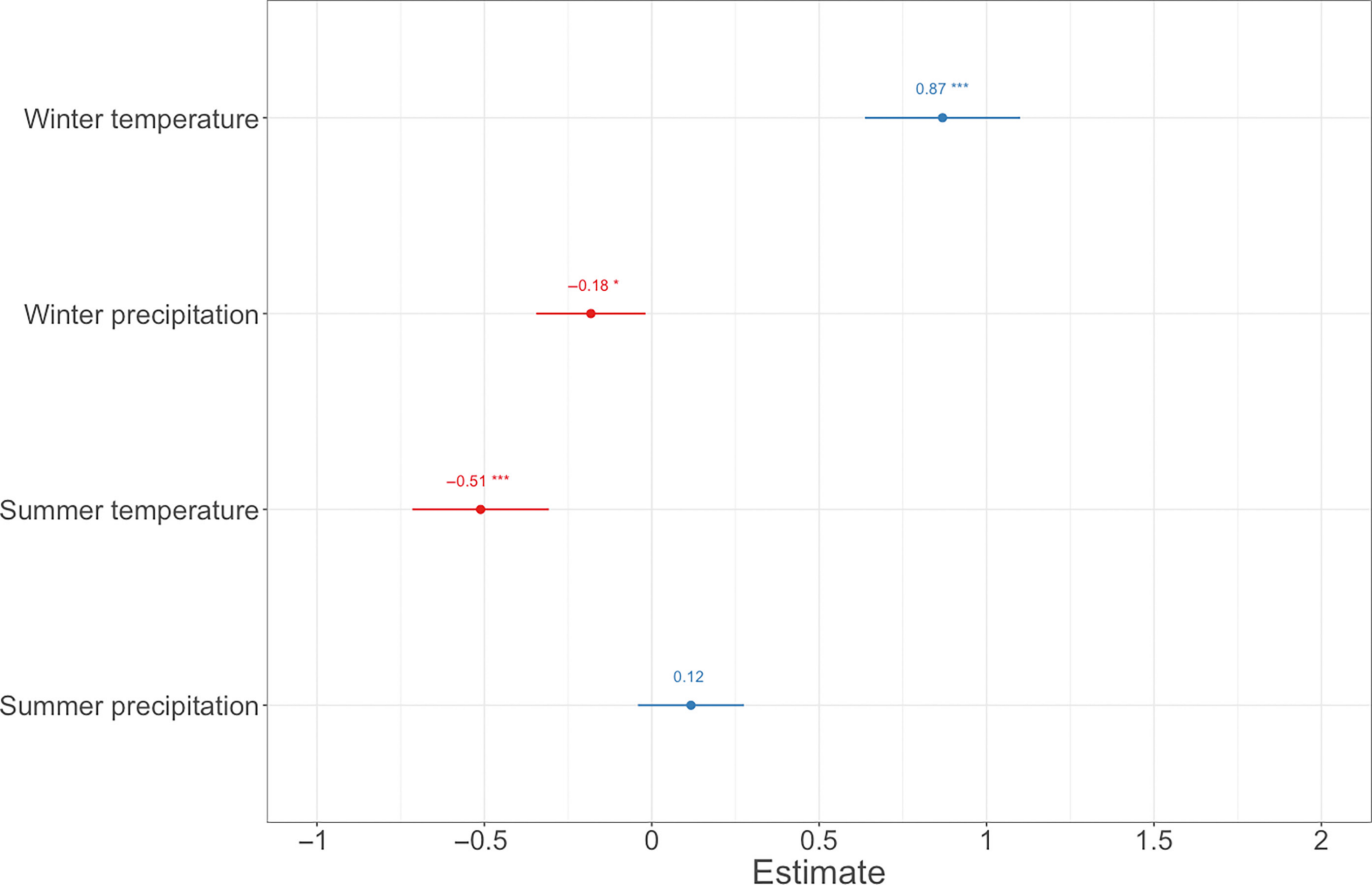
Variable estimates of GLMER statistics of a model explaining the probability of an owl being brown (1900–2016) with general climatic conditions (weather variables as means of 1950–2016). Weather variables are standardised to mean value ±1 SD. Asterisks indicate the significance level of the *p*‐values (*** = <.001, ** = <.01, * = <.05).

**FIGURE 5:**
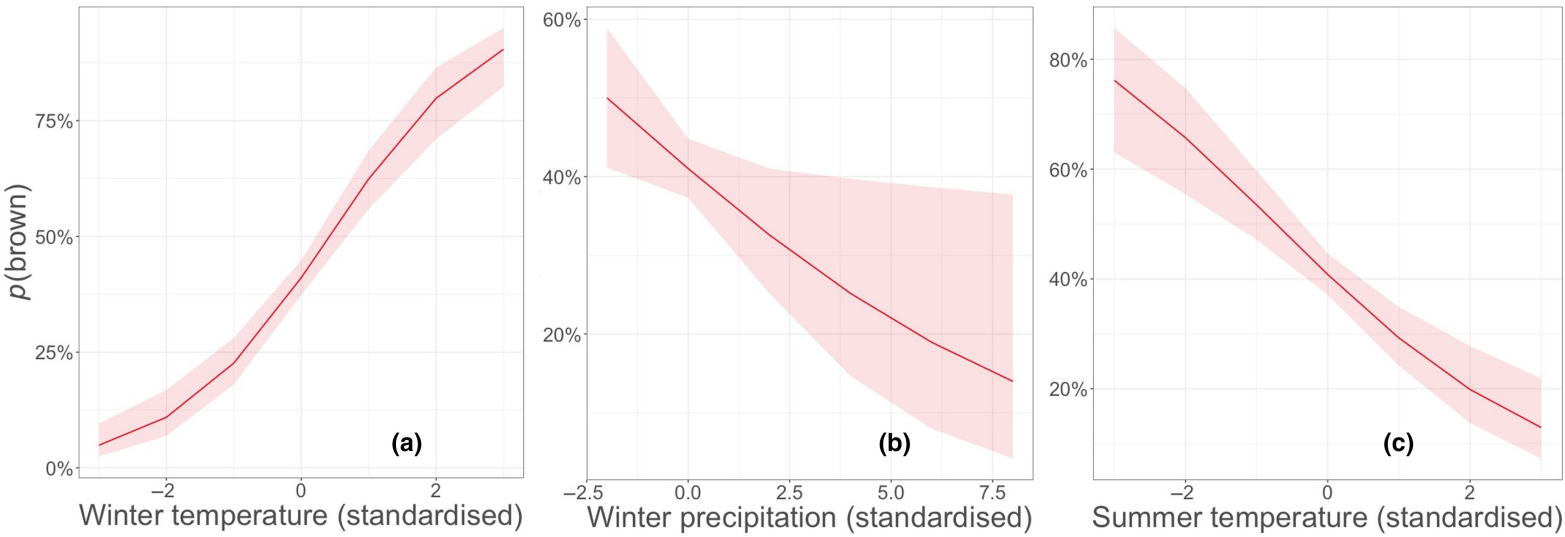
*p*(brown) explained by standardised (a) winter temperature, (b) winter precipitation and (c) summer temperature (estimates ± CI of the GLMER) according to general climatic conditions model (Table S2).

There was a strong positive connection between *p*(brown) and the long‐term mean winter temperature (Figures 4 and 5a) and a negative connection with winter precipitation (Figures 4 and 5b). Long‐term mean summer temperature was negatively associated with *p*(brown) (Figures 4 and 5c), whereas there was no association between summer precipitation and *p*(brown) (Figure 4). Full model output is shown in Table S2. The models ran separately for each climate zone support the main findings (Table S3).

### 3.3 Associations between *p*(brown) and short‐term climatic conditions, 1955–2016

**FIGURE 6:**
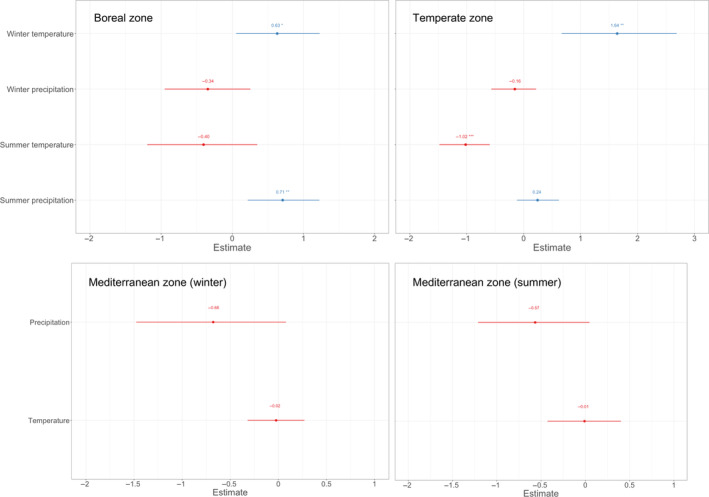
Variable estimates of GLM statistics of models explaining the probability of an owl being brown (1955–2016) with 5‐year means of weather variables prior to the observation of an owl on each climate zone. Asterisks indicate the significance level of the *p*‐values (*** = <.001, ** = <.01, * = <.05).

In boreal zone, both the mean summer precipitation and mean winter temperatures 5 years prior to the observation were positively associated with *p*(brown) (Figure 6, Boreal zone). In the temperate zone, mean winter temperature prior to observation was also positively associated with *p*(brown), whereas mean summer temperature prior to observation was negatively associated with *p*(brown) (Figure 6, Temperate zone). In the Mediterranean zone, there was a tendency for a negative association between both winter temperature prior to observation and *p*(brown) and mean summer temperature prior to observation and *p*(brown) (Figure 6, Mediterranean zone). Full model outputs are shown in Table S4. Results of 1‐ and 3‐year means models were qualitatively similar (see Tables S5 and S6 for statistics).

## 4 DISCUSSION

We found that in tawny owls, which display a pheomelanic brown and pale grey morph, the probability that the observed individuals are brown is largely explained by the climatic zone and the year they are observed. In addition, we found support that general climatic conditions (long‐term means of weather) dictate the spatial distribution. We found that the probability to observe a brown morph was higher in areas with higher mean winter temperatures and lower mean winter precipitation, and lower in areas with higher summer temperatures. Furthermore, 5‐year means of weather prior to observation affected the probability to observe a brown morph differently between climate zones. In the boreal zone winter temperature prior to the observations were positively associated with the probability that the owls were brown. In addition, summer precipitation was positively associated with *p*(brown) in the boreal zone. Similarly, in the temperate zone winter temperature was positively associated, and summer temperature was negatively associated with the probability that the owl was brown. In the Mediterranean zone, winter temperature tended to have an opposite effect on *p*(brown) whereas lower summer temperatures had a similar positive effect on the probability that the owl was brown.

In our historical (museum skin colouration) data spanning more than 100 years, the brown morph seems to have dominated in every climate zone in the early 1900s (Figure 3). However, temporal changes in colour morph frequencies were detected within the climate zones. The colouration of owls shifted over time towards grey in the temperate‐ and Mediterranean zone. The situation in the boreal zone is more complex, as the frequency of brown morph seemed to decline at first but increased again during the mid‐1900s (Figure 3). This supports our main hypothesis that climate change has altered the occurrence of colour morphs according to the predictions of the simple version of Gloger's rule. This is because the frequency of brown morph has increased in the boreal zone that has faced strong warming and increased precipitation (EEA, 2017), whereas frequencies of brown owls have decreased in the Mediterranean zone which has faced decreasing precipitation (EEA, 2017). The decrease in brown morph frequency in the temperate zone is harder to explain in the context of this study, as this zone has not faced as strong directional climatic changes as the other two climate zones (EEA, 2017).

The strongest association between *p*(brown) and a long‐term mean of a weather variable was with winter temperature. The finding is in accordance with Gloger's rule as warm winters were associated with an increase in *p*(brown). On the contrary, warm summers were associated with a decrease in *p*(brown). In many parts of Europe, warm summers are also dry, which could explain the result. However, there was no association between summer precipitation and *p*(brown). Areas with high winter precipitation were associated with a low probability to observe a brown owl, which contrasts Gloger's rule. However, increasing winter precipitation can indicate snow cover in many parts of the boreal zone, which can favour grey morph over brown regarding camouflage functions in snowy landscapes (Koskenpato et al., 2020). It seems evident that the boreal zone is currently experiencing the greatest change, both regarding climate and the colouration of tawny owl colour morphs. Winters have traditionally been snowy and cold in the boreal zone, but since the 1960s the zone has faced increasing winter temperatures and precipitation, and the trend is predicted to continue with increasing intensity (EEA, 2017). It is already documented with population monitoring data that the brown tawny owl morph has increased in frequency in the species northernmost range margin in Finland since the 1960s because of improved winter survival as the winters have gotten milder with higher temperatures and less snow (Karell et al., 2011). Increased snow depth can hamper survival, especially of the brown morph (Karell et al., 2011) but milder winters with higher rainfall (Hurrell, 1995; when snow is replaced by rain) can improve the survival of brown.

Differing temporal trends of *p*(brown) between climate zones can be partly explained by climate zone‐specific weather effects, as 5‐year means of weather prior to observation explained the probability that the owl was brown, especially in the boreal and the temperate zone. As in the general climatic conditions model, the mean winter temperature 5 years prior to observation was also positively associated with *p*(brown) in the boreal and temperate zones. Considering the recent increase in temperature faced especially during winters (EEA, 2017), these results combined support previous findings of low survival of brown owls in harsh winters (Karell et al., 2011). In addition, hot summers 5 years before observations also predicted low *p*(brown) in the temperate zone (with also tendency in the Mediterranean zone), thus in line with the effect of summer temperatures we observed in the general climatic conditions model. According to Gloger's rule, increasing temperature favours melanistic individuals, but hot summers may also be dry, which can in turn favour less melanistic morphs with marked effect at least in the generally humid temperate zone. Summer precipitation had no connection with *p*(brown) in the general climatic conditions model but was positively associated with *p*(brown) in the 5‐year means model of the boreal zone.

In addition to climate, other anthropogenic stressors likely affect the temporal trends observed in tawny owl colouration patterns in Europe. A recent review (Sumasgutner et al., 2023) highlights that climate change can interact with other abiotic and biotic factors and that the outcomes of these interactions can be climate‐zone‐specific. Habitat fragmentation is considered to be one of the main phenomena altering species existence (Ewers & Didham, 2006). Urbanisation‐related changes in luminal conditions may affect the occurrence of colour morphs if the morphs are adapted differently, for example by means of camouflage (Gehlbach, 1994; Passarotto et al., 2018). Also, local climate conditions may differ as urban areas are in general warmer than rural areas (Tzavali et al., 2015), further leading to differences in, for example snow cover and thus camouflage functions (Koskenpato et al., 2020). What truly shapes the occurrence of tawny owl colour morphs is probably a combination of many factors. Studies have suggested, for example effects of light conditions and habitat structure on the occurrence of colour polymorphism and colouration in birds (Galeotti et al., 2003; Gehlbach, 1994; Passarotto et al., 2018, 2021). However, although we fully acknowledge that local scale effects of habitat and biotic interactions are important drivers of colour polymorphism in the tawny owl and other colour polymorphic species, we believe that we have here shown some fundamental parts of the big picture of colour morph distribution in this species.

There are many subcategories in the Köppen‐Geiger climate classification (Rubel & Kottek, 2010) and in our model, we have pooled some of them together to represent broader climate zones to reduce heterogeneity and add statistical power. We further justify the combining of these subcategories with the Köppen‐Geiger climate classification scheme (Rubel & Kottek, 2010) where the subcategories used in this study are defined to group together accordingly. Still, the microhabitats within a single zone may differ and even have opposing effects on the colour morph occurrence (e.g. humidity and vegetation). This is something we are unable to detect within the context of this study. It has recently been discussed that old ecogeographical rules, such as Gloger's rule, may not hold as initially predicted in the prevailing environment facing climate change and instead of geographical location, the micro‐environments probably have a stronger effect on shaping the phenotypes of organisms (Goldenberg et al., 2022). However, our approach to explaining tawny owl colour morph frequency with climate zones, long‐term weather means and short‐term weather means enables detecting the complexity of potential climate‐associated selection pressures operating on colour morphs.

As tawny owls are highly territorial year‐round (Saurola, 1995; Sunde, 2012), the observed locations of owl specimens used in this study very likely represent the sites where the owls live and breed in nature. From a geographical point of view, the data of owl specimens are not evenly distributed inside the climate zones (Figure 1). In the boreal zone, most of the data are located in southern Finland and northern Italy around the Alps. In the temperate zone, the data are mostly located in the United Kingdom, northern Italy and southern Sweden. In the Mediterranean zone, the data are more scattered. Maybe not surprisingly, these hot spots are located around the museums where the data were gathered from. By using the method of studying museum specimens, the uneven distribution of data seems inevitable. However, the overrepresented areas are distributed well inside the climate zones. Southern Finland and northern Italy are on opposite sides of the whole boreal zone. Also, the United Kingdom, northern Italy and southern Sweden are distributed well inside the temperate zone. We, therefore, believe our data represents a sample from various parts of each of the climate zones.

We cannot rule out that one or the other colour morph would be overrepresented in the museum collections due to a bias in sampling (Cooper et al., 2019). However, we believe it is unlikely that one of the morphs would appear more attractive to collectors and thus be gathered more eagerly as a museum specimen. The cause of death of an owl is rarely provided by the museums, but it is unlikely that an owl is selected based on colour and then killed by a human for a museum collection. Some of the owl specimens could have been starved to death, potentially because of the weather. In that case, we would expect a higher proportion of the non‐predicted morph among those samples (Galeotti & Cesaris, 1996). There is no evidence that either of the tawny owl morphs would prefer urban areas to such an extent that it would affect the finding probability of dead owls of a certain morph. In a study by Galeotti and Cesaris (1996), the authors stated that the majority (73%) of museum specimens used in the study were starved or died for unknown reasons, and a minority (27%) were shot. It is also discussed, that regarding museum specimens, the sampling protocols of museums may change over time along curators and that sample sizes can vary remarkably among years (Schroeder et al., 2009). This variation in sampling is inherent also in our data set, however, we have no reason to believe that any sampling bias would be biased to either morph. Since we are interested in the relative proportions of the morphs, we feel our analyses are robust to this variation. Therefore, museum collections enable investigations of variation in traits over long time periods and can be useful to capture evolutionary trends over time (Holmes et al., 2016), such as our analyses of variation in highly heritable colour morph distributions in time and space.

The mechanisms underlying the detected evolutionary patterns using museum collections may be hard or even impossible to identify. Here, we are quite confident that climate change plays a great role in shaping the distribution patterns of tawny owl colour morphs across Europe. This is due to our understanding of the tawny owl system based on previous findings (Galeotti & Cesaris, 1996; Gasparini et al., 2009; Karell et al., 2011; Koskenpato et al., 2016, 2020) and the predictions of Gloger's rule (Delhey, 2017, 2019). To conclude, tawny owl colouration seems not to purely follow Gloger's rule, and there are likely other factors involved in shaping the distribution of colour morphs (e.g. camouflage functions in snowy winters). The most dominant factors affecting colouration probably vary in time and space. Overall, our results support previous findings (Galeotti & Cesaris, 1996; Karell et al., 2011; Koskenpato et al., 2016; Piault et al., 2009) suggesting that the grey morph is better adapted to extreme conditions (e.g. cold and snowy winters, and hot summers), whereas the brown morph seems to do better in moderate conditions. As tawny owl colouration is highly heritable (Brommer et al., 2005; Gasparini et al., 2009; Karell et al., 2011; Morosinotto et al., 2020), we can consider the colouration as a proxy of genetic variation. Thus, our results indicate that there are shifts in genotype frequencies that can be at least partly because of changes in the climate and that the genetic variation is changing markedly in these climate zones across over a century. Also, the different climate zones may serve as barriers to gene flow. As colour morphs are considered to be adaptations to different environmental conditions (Roulin, 2004), they are useful proxies for environmental change. Our study adds a spatiotemporal complement to otherwise scarce studies linking microevolutionary patterns associated with climate change (Gienapp et al., 2008) and the emergence of Gloger's rule (Delhey, 2019; Delhey et al., 2020; Passarotto et al., 2021).

## AUTHOR CONTRIBUTIONS

**Katja Koskenpato:** Conceptualization (lead); data curation (equal); formal analysis (equal); methodology (equal); validation (equal); writing – original draft (lead); writing – review and editing (equal). **Aleksi Lehikoinen:** Conceptualization (supporting); data curation (equal); formal analysis (equal); methodology (equal); validation (equal); writing – review and editing (equal). **Chiara Morosinotto:** Conceptualization (supporting); data curation (equal); methodology (equal); validation (equal); writing – review and editing (equal). **Ruslan Gunko:** Data curation (equal); methodology (supporting); writing – review and editing (equal). **Patrik Karell:** Conceptualization (lead); data curation (equal); formal analysis (equal); methodology (equal); validation (equal); writing – review and editing (equal).

## CONFLICT OF INTEREST STATEMENT

The authors do not have any conflict of interest.

## Supporting information


Data S1:

## Data Availability

All data and codes can be found in Dryad: https://doi.org/10.5061/dryad.kkwh70s92.
