# Supplementary material for: Regional variation in climate change alters the range‐wide distribution of colour polymorphism in a wild bird
Source: Ecol Evol. 2023 Jul 17;13(7):e10311. doi: 10.1002/ece3.10311 (PMC10352091; doi:10.1002/ece3.10311)
Supplement: Supplementary file 1 — Data S1: [file ECE3-13-e10311-s001.docx]

SUPPLEMENTARY MATERIAL FOR:

**Regional variation in climate change alters the range-wide distribution of colour polymorphism in a wild bird**

List of the institutions that provided tawny owl specimens for this study:

1. Prague Natural History Museum, Czech Republic
2. Finnish Museum of Natural History, Helsinki, Finland
3. Kuopio Natural History Museum, Kuopio, Finland
4. Natural History Museum Berlin, Germany
5. Natural History Museum of Carmagnola, Italy
6. Natural History Museum of Genova, Italy
7. Natural History Museum of Milano, Italy
8. Natural History Museum of Venezia, Italy
9. Natural History Museum of Verona, Italy
10. Zoological Museum of Roma, Italy
11. museum within the “Istituto Superiore per la Protezione e la Ricerca Ambientale”, ISPRA, Italy
12. Science Museum in Trento (MUSE), Italy
13. Zoological Museum of St Petersburg, Russia
14. Zoological Museum of Moscow, Russia
15. Estacion Biologica de Doñana, Spain
16. Museum Nacional de Ciencias Naturales MNCN, Spain
17. Biological Museum at Lund University, Sweden
18. University Museum of Zoology, Cambridge, UK
19. Natural History Museum of Tring, UK

Supplementary table 1. GLM statistics of a model explaining the temporal patterns in the probability of an owl being brown (1900–2016) with climate zones. MZ = Mediterranean zone, BZ = boreal zone. Temperate zone is the reference category. Significant values (p < 0.05) are shown in bold.

|  | Estimate | Std. Error | z value | p value |
| --- | --- | --- | --- | --- |
| Intercept | 0.118 | 0.107 | 1.101 | 0.271 |
| Year | **-18.686** | **3.518** | **-5.312** | **<0.001** |
| Year^2^ | 0.994 | 3.337 | 0.298 | 0.766 |
| MZ | **-0.385** | **0.195** | **-1.972** | **0.049** |
| BZ | **-0.928** | **0.145** | **-6.398** | **<0.001** |
| MZ * Year | 12.476 | 6.829 | 1.827 | 0.068 |
| MZ * Year^2^ | -0.292 | 6.183 | -0.047 | 0.962 |
| BZ * Year | **11.196** | **4.642** | **2.412** | **0.016** |
| BZ * Year^2^ | 9.094 | 4.712 | 1.930 | 0.054 |
|  |  |  |  |  |

Supplementary table 2. GLMER statistics of a model explaining the probability of an owl being brown (1900–2016) with general climatic conditions (weather variables as means of 1950–2016). Weather variables are standardised to mean value ± 1 SD. Significant values (p < 0.05) are shown in bold.

|  | Estimate | | Std. Error | z value | p value |
| --- | --- | --- | --- | --- | --- |
| Intercept | **-0.368** | **0.079** | | **-4.650** | **<0.001** |
| Winter temperature | **0.869** | **0.118** | | **7.342** | **<0.001** |
| Winter precipitation | **-0.182** | **0.083** | | **-2.186** | **0.029** |
| Summer temperature | **-0.511** | **0.104** | | **-4.917** | **<0.001** |
| Summer precipitation | 0.117 | 0.081 | | 1.447 | 0.148 |

Supplementary table 3. GLMER statistics of models explaining the probability of an owl being brown (1900–2016) with general climatic conditions (weather variables as means of 1950–2016) in each climate zone. Weather variables are standardised to mean value ± 1 SD. Significant values (p < 0.05) are shown in bold. Due to high correlation between some variables in Mediterranean zone, we conducted separate models for winter and summer climate. In all cases, variance of the random effect *Year* was zero and thus models were converted to run as GLMs.

|  | Estimate | Std. Error | z value | p value |
| --- | --- | --- | --- | --- |
| ***Boreal zone*** |  |  |  |  |
| Intercept | **-1.000** | **0.302** | **-3.314** | **<0.001** |
| Winter temperature | 0.025 | 0.260 | 0.095 | 0.923 |
| Winter precipitation | -0.517 | 0.355 | -1.455 | 0.146 |
| Summer temperature | 0.331 | 0.238 | 1.390 | 0.164 |
| **Summer precipitation** | **0.593** | **0.187** | **3.181** | **0.001** |
| ***Temperate zone*** |  |  |  |  |
| Intercept | **-0.900** | **0.254** | **-3.539** | **<0.001** |
| Winter temperature | **2.121** | **0.434** | **4.887** | **<0.001** |
| Winter precipitation | **-0.443** | **0.151** | **-2.936** | **0.003** |
| Summer temperature | **-0.934** | **0.162** | **-5.785** | **<0.001** |
| Summer precipitation | 0.227 | 0.143 | 1.582 | 0.114 |
| ***Mediterranean zone winter*** |  |  |  |  |
| Intercept | 0.388 | 0.573 | 0.677 | 0.498 |
| Winter temperature | -0.463 | 0.341 | -1.360 | 0.174 |
| Winter precipitation | -0.028 | 0.141 | -0.196 | 0.844 |
| ***Mediterranean zone summer*** |  |  |  |  |
| Intercept | 0.209 | 0.327 | 0.640 | 0.522 |
| Summer temperature | -0.455 | 0.275 | -1.657 | 0.098 |
| Summer precipitation | -0.074 | 0.209 | -0.353 | 0.724 |

|  |  |  |  |  |
| --- | --- | --- | --- | --- |

Supplementary table 4. GLM statistics of models explaining the probability of an owl being brown (1955–2016) with five-year means of weather variables prior the observation of an owl. Significant values (p < 0.05) are shown in bold.

|  | Estimate | Std. Error | z value | p value |
| --- | --- | --- | --- | --- |
| ***Boreal zone*** |  |  |  |  |
| Intercept | **-1.161** | **0.338** | **-3.432** | **<0.001** |
| Winter temperature | **0.631** | **0.298** | **2.116** | **0.034** |
| Winter precipitation | -0.344 | 0.306 | -1.125 | 0.261 |
| Summer temperature | -0.404 | 0.392 | -1.032 | 0.302 |
| Summer precipitation | **0.708** | **0.255** | **2.772** | **0.006** |
| ***Temperate zone*** |  |  |  |  |
| Intercept | **-0.906** | **0.290** | **-3.126** | **0.002** |
| Winter temperature | **1.642** | **0.513** | **3.200** | **0.001** |
| Winter precipitation | -0.156 | 0.197 | -0.791 | 0.429 |
| Summer temperature | **-1.020** | **0.225** | **-4.541** | **<0.001** |
| Summer precipitation | 0.243 | 0.186 | 1.308 | 0.191 |
| ***Mediterranean zone winter*** |  |  |  |  |
| Intercept | 0.565 | 0.681 | 0.829 | 0.407 |
| Winter temperature | -0.677 | 0.395 | -1.716 | 0.086 |
| Winter precipitation | -0.023 | 0.149 | -0.155 | 0.877 |
| ***Mediterranean zone summer*** |  |  |  |  |
| Intercept | 0.331 | 0.394 | 0.840 | 0.401 |
| Summer temperature | -0.567 | 0.318 | -1.783 | 0.075 |
| Summer precipitation | -0.009 | 0.210 | -0.043 | 0.965 |

Supplementary table 5. GLM statistics of models explaining the probability of an owl being brown (1955–2016) with one-year mean of weather variables prior the observation of an owl. Significant values (p < 0.05) are shown in bold.

|  | Estimate | Std. Error | z value | p value |
| --- | --- | --- | --- | --- |
| ***Boreal zone*** |  |  |  |  |
| Intercept | **-1.049** | **0.246** | **-4.265** | **<0.001** |
| Winter temperature | **0.526** | **0.235** | **2.236** | **0.025** |
| Winter precipitation | -0.320 | 0.241 | -1.328 | 0.184 |
| Summer temperature | -0.487 | 0.315 | -1.547 | 0.122 |
| Summer precipitation | 0.162 | 0.170 | 0.954 | 0.340 |
| ***Temperate zone*** |  |  |  |  |
| Intercept | **-0.566** | **0.258** | **-2.198** | **0.028** |
| Winter temperature | **0.918** | **0.422** | **2.177** | **0.030** |
| Winter precipitation | -0.252 | 0.186 | -1.359 | 0.174 |
| Summer temperature | **-0.764** | **0.193** | **-3.962** | **<0.001** |
| Summer precipitation | -0.049 | 0.147 | -0.331 | 0.741 |
| ***Mediterranean zone winter*** |  |  |  |  |
| Intercept | 0.747 | 0.609 | 1.228 | 0.220 |
| Winter temperature | **-0.870** | **0.407** | **-2.135** | **0.033** |
| Winter precipitation | -0.037 | 0.144 | -0.260 | 0.795 |
| ***Mediterranean zone summer*** |  |  |  |  |
| Intercept | 0.319 | 0.392 | 0.812 | 0.417 |
| Summer temperature | -0.486 | 0.292 | -1.662 | 0.097 |
| Summer precipitation | 0.093 | 0.234 | 0.400 | 0.690 |

Supplementary table 6. GLM statistics of models explaining the probability of an owl being brown (1955–2016) with three-year means of weather variables prior the observation of an owl. Significant values (p < 0.05) are shown in bold.

|  | Estimate | Std. Error | z value | p value |
| --- | --- | --- | --- | --- |
| ***Boreal zone*** |  |  |  |  |
| Intercept | **-1.065** | **0.297** | **-3.587** | **<0.001** |
| Winter temperature | **0.625** | **0.273** | **2.295** | **0.022** |
| Winter precipitation | -0.294 | 0.278 | -1.058 | 0.2900 |
| Summer temperature | -0.384 | 0.373 | -1.038 | 0.299 |
| Summer precipitation | **0.543** | **0.223** | **2.433** | **0.015** |
| ***Temperate zone*** |  |  |  |  |
| Intercept | **-0.783** | **0.292** | **-2.684** | **0.007** |
| Winter temperature | **1.351** | **0.496** | **2.725** | **0.006** |
| Winter precipitation | -0.162 | 0.195 | -0.833 | 0.405 |
| Summer temperature | **-0.902** | **0.208** | **-4.330** | **<0.001** |
| Summer precipitation | 0.165 | 0.172 | 0.964 | 0.335 |
| ***Mediterranean zone winter*** |  |  |  |  |
| Intercept | 0.704 | 0.687 | 1.025 | 0.306 |
| Winter temperature | -0.806 | 0.416 | -1.939 | 0.053 |
| Winter precipitation | -0.015 | 0.149 | -0.100 | 0.921 |
| ***Mediterranean zone summer*** |  |  |  |  |
| Intercept | 0.332 | 0.395 | 0.840 | 0.401 |
| Summer temperature | -0.528 | 0.309 | -1.708 | 0.088 |
| Summer precipitation | 0.033 | 0.214 | 0.156 | 0.876 |


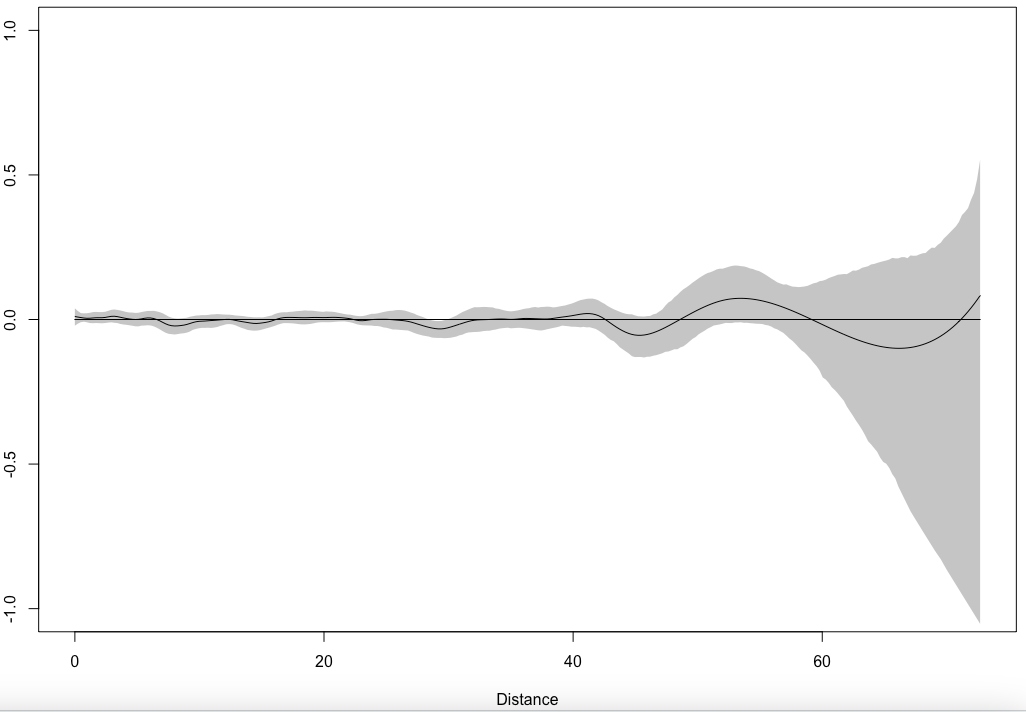


Supplementary figure 1. Spatial autocorrelation correlogram based on residuals of **climate zone model** (*colour morph ~ year * climate zone + year^2^ * climate zone*) (Table 1).


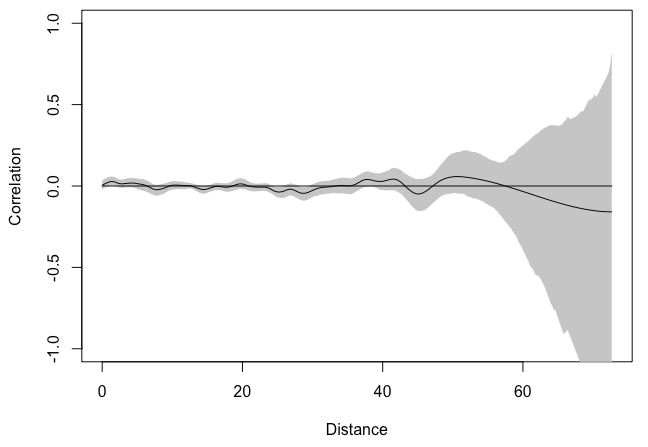


Supplementary figure 2. Spatial autocorrelation correlogram based on residuals of **general climatic conditions model** (*colour morph ~ 1|year + winter temperature + winter precipitation + summer temperature + summer precipitation* ) (Table 2).


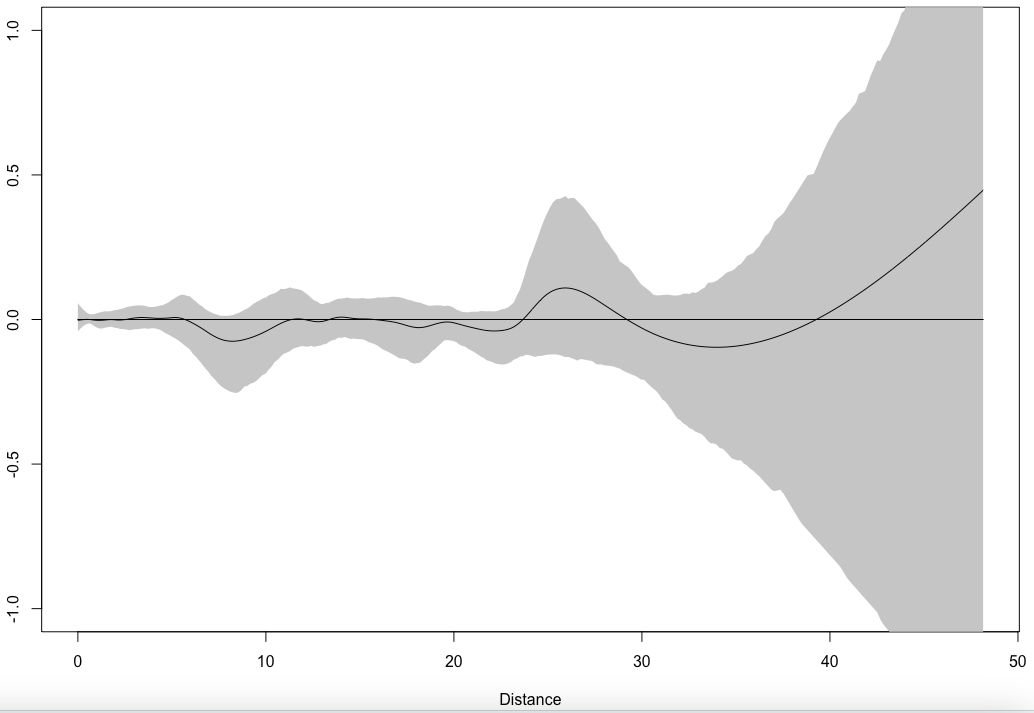


Supplementary figure 3. Spatial autocorrelation correlogram based on residuals of **boreal zone five-year means model** (*colour morph ~ 5yr mean winter temperature + 5yr mean winter precipitation + 5yr mean summer temperature + 5yr mean summer precipitation*) (Table 3, boreal zone).


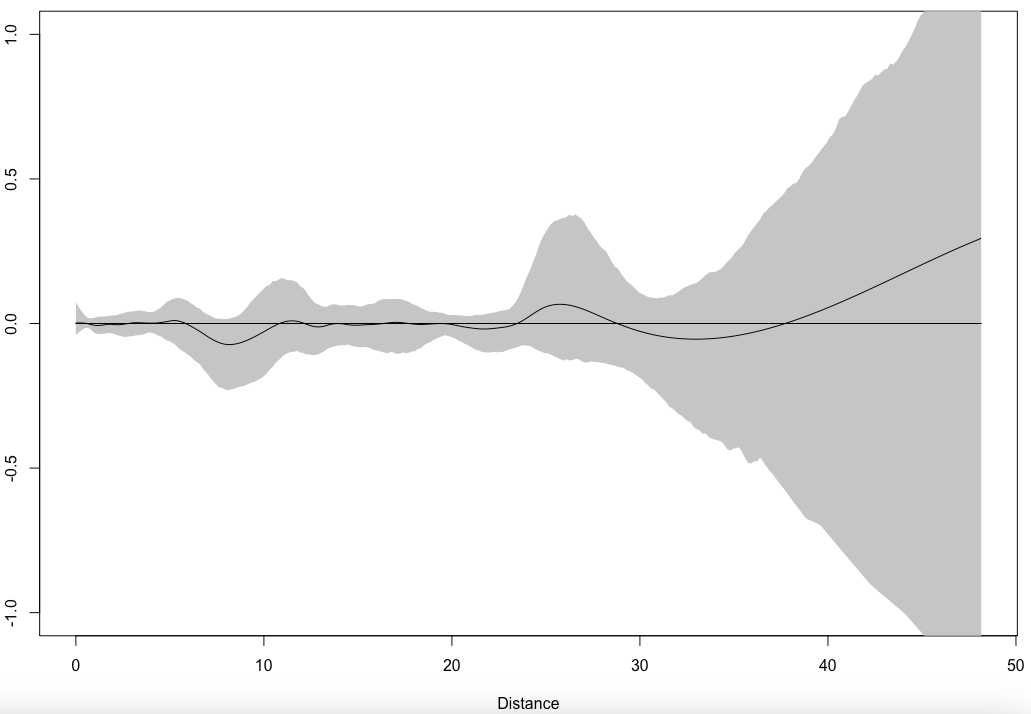


Supplementary figure 4. Spatial autocorrelation correlogram based on residuals of **temperate zone five-year means model** (*colour morph ~ 5yr mean winter temperature + 5yr mean winter precipitation + 5yr mean summer temperature + 5yr mean summer precipitation*) (Table 3, temperate zone).


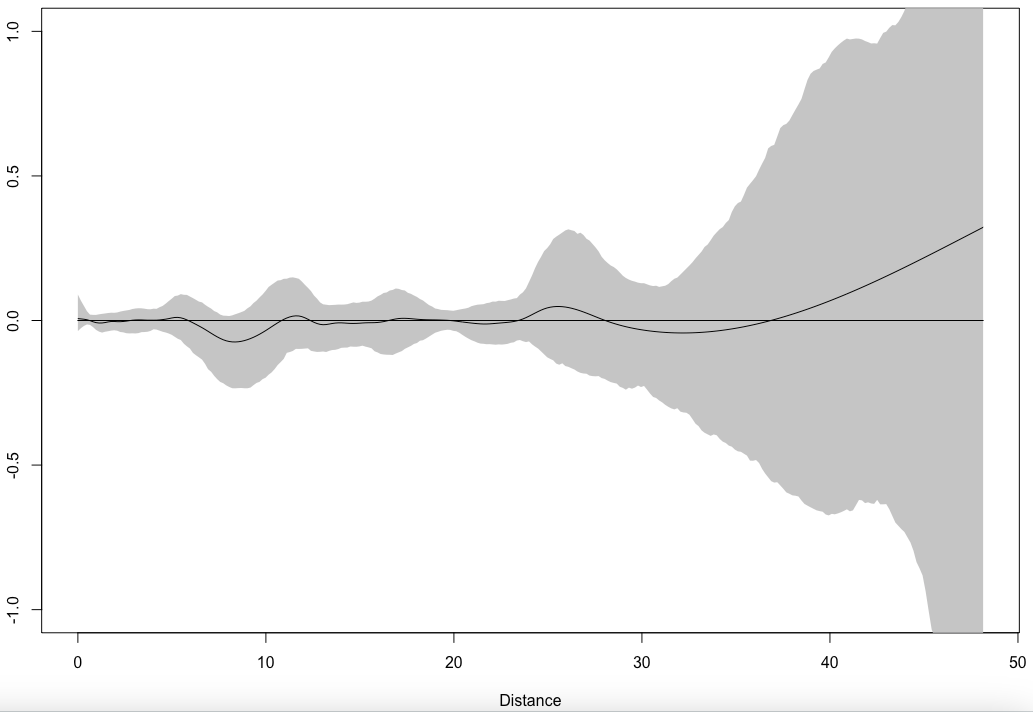


Supplementary figure 5. Spatial autocorrelation correlogram based on residuals of **Mediterranean zone winter five-year means model** (*colour morph ~ 5yr mean winter temperature + 5yr mean winter precipitation*) (Table 3, Mediterranean zone winter).


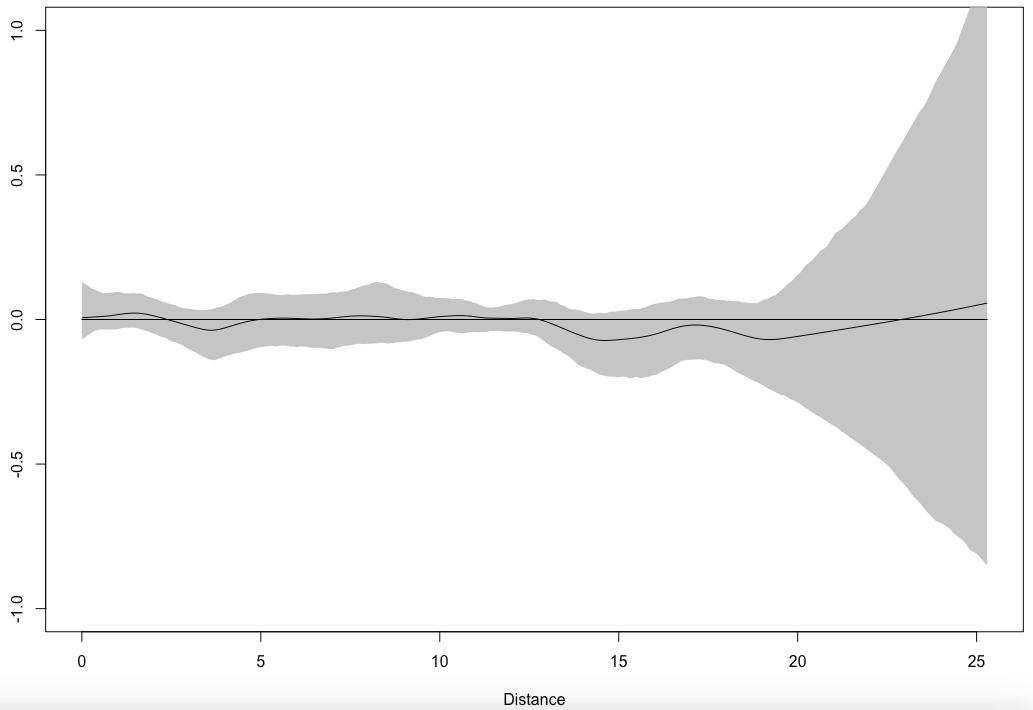


Supplementary figure 6. Spatial autocorrelation correlogram based on residuals of **Mediterranean zone summer five-year means model** (*colour morph ~ 5yr mean summer temperature + 5yr mean summer precipitation*) (Table 3, Mediterranean zone summer).


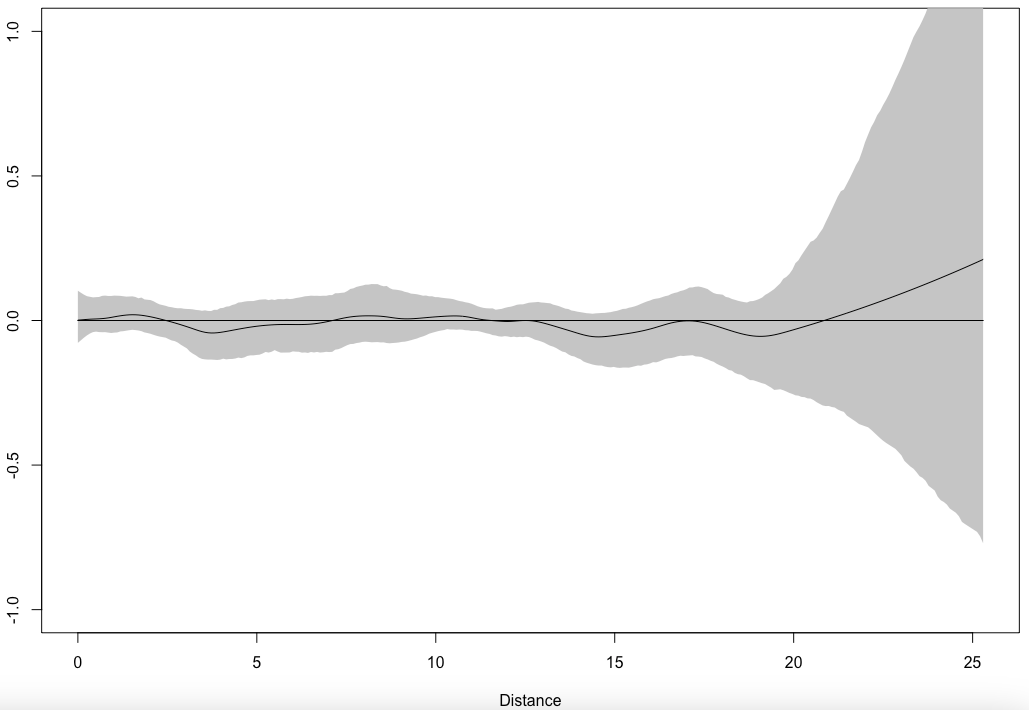


Supplementary figure 7. Spatial autocorrelation correlogram based on residuals of **boreal zone three-year means model** (*colour morph ~ 3yr mean winter temperature + 3yr mean winter precipitation + 3yr mean summer temperature + 3yr mean summer precipitation*) (Supplementary table 1, boreal zone).


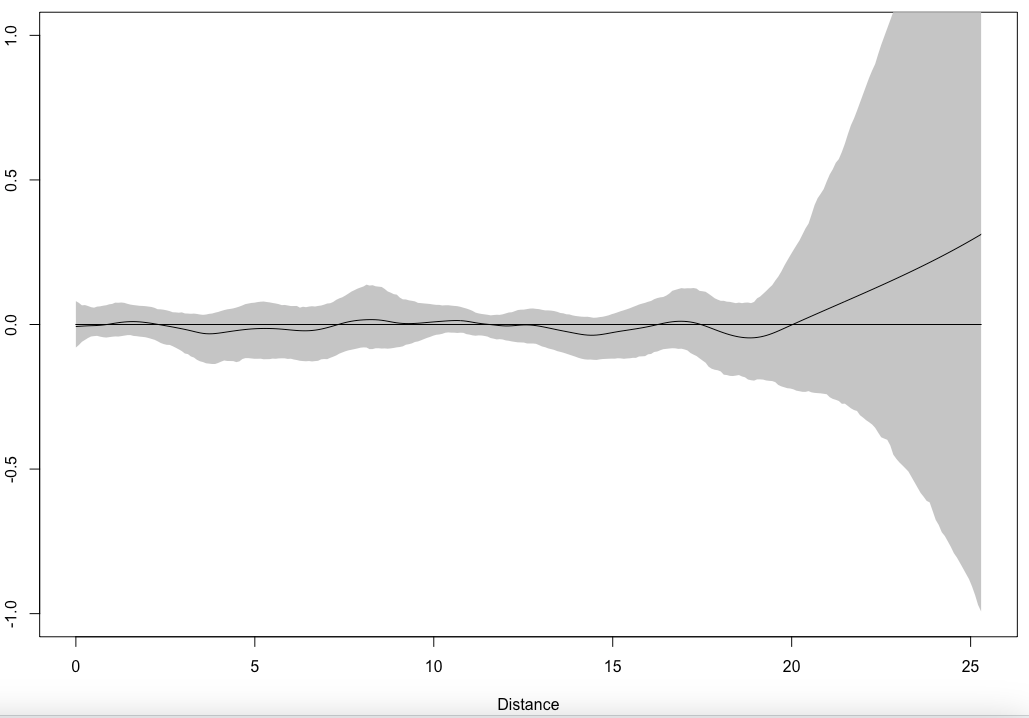


Supplementary figure 8. Spatial autocorrelation correlogram based on residuals of **temperate zone three-year means model** (*colour morph ~ 3yr mean winter temperature + 3yr mean winter precipitation + 3yr mean summer temperature + 3yr mean summer precipitation*) (Supplementary table 1, temperate zone).

**
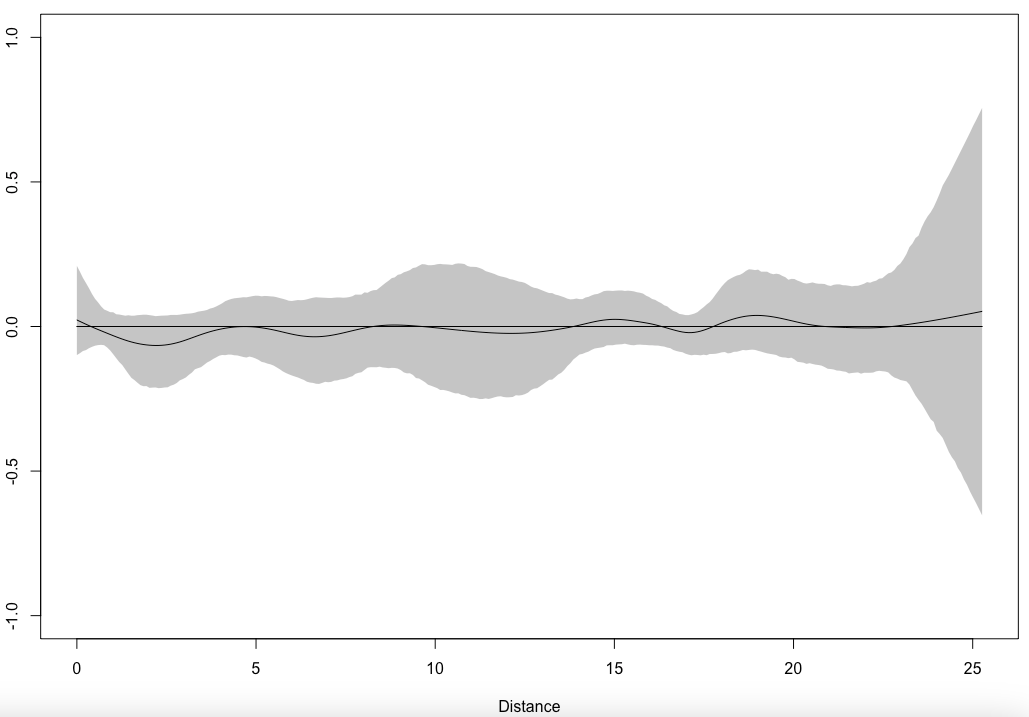
**

Supplementary figure 9. Spatial autocorrelation correlogram based on residuals of **Mediterranean zone winter three-year means model** (*colour morph ~ 3yr mean winter temperature + 3yr mean winter precipitation*) (Supplementary table 1, Mediterranean zone winter).


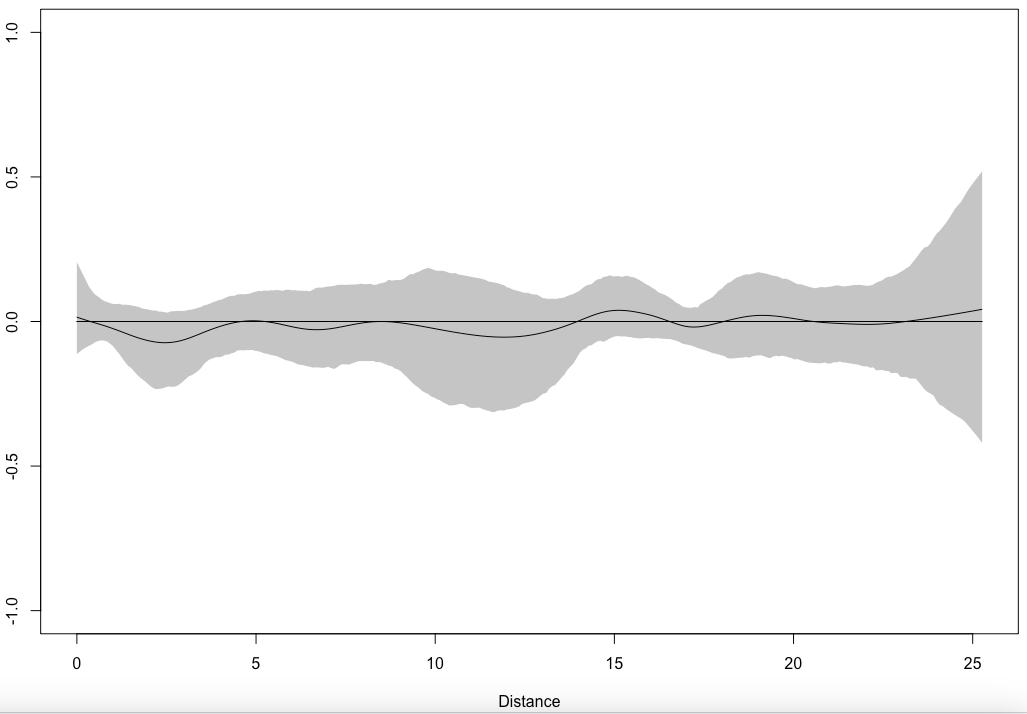


Supplementary figure 10. Spatial autocorrelation correlogram based on residuals of **Mediterranean zone summer three-year means model** (*colour morph ~ 3yr mean summer temperature + 3yr mean summer precipitation*) (Supplementary table 1, Mediterranean zone summer).


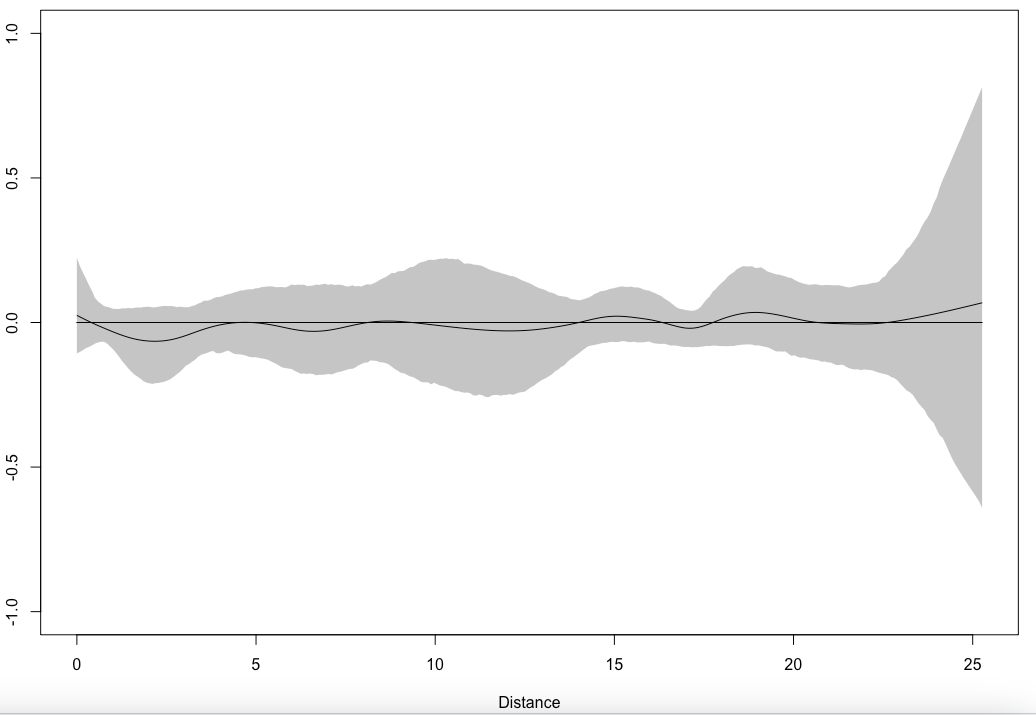


Supplementary figure 11. Spatial autocorrelation correlogram based on residuals of **boreal zone one-year mean model** (*colour morph ~ 1yr mean winter temperature + 1yr mean winter precipitation + 1yr mean summer temperature + 1yr mean summer precipitation*) (Supplementary table 2, boreal zone).


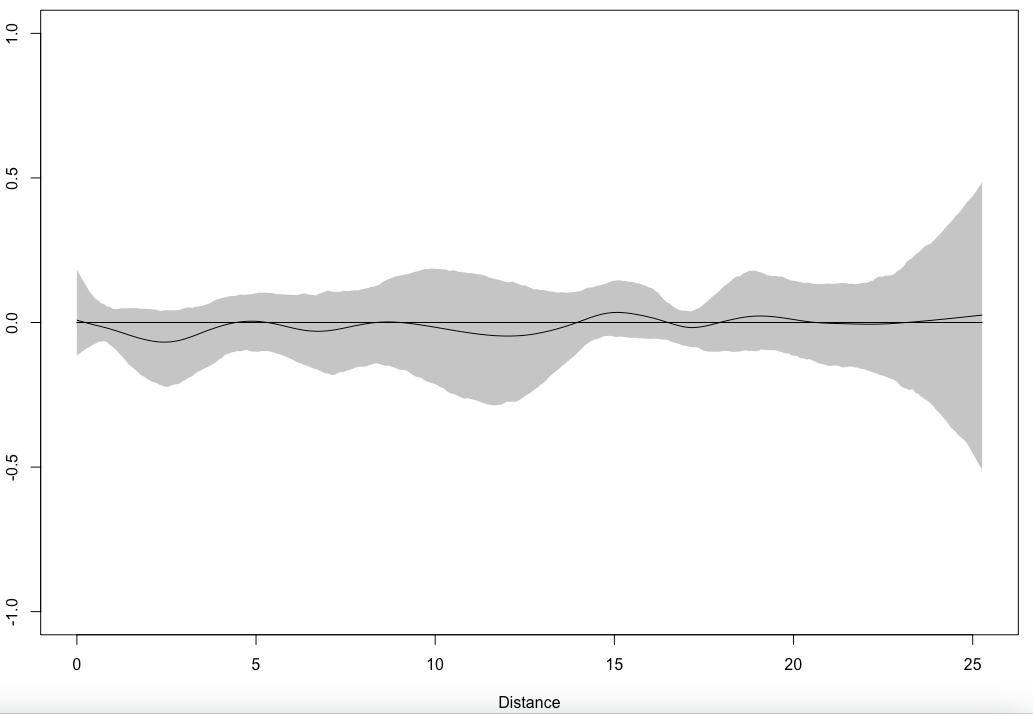


Supplementary figure 12. Spatial autocorrelation correlogram based on residuals of **temperate zone one-year mean model** (*colour morph ~ 1yr mean winter temperature + 1yr mean winter precipitation + 1yr mean summer temperature + 1yr mean summer precipitation*) (Supplementary table 2, temperate zone).


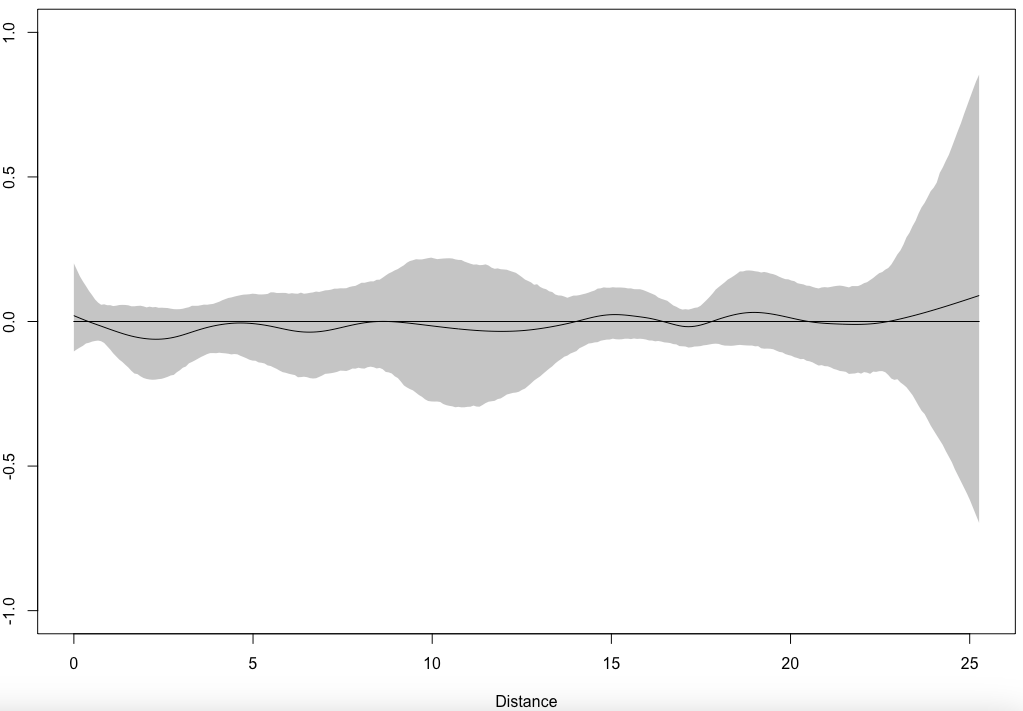


Supplementary figure 13. Spatial autocorrelation correlogram based on residuals of **Mediterranean zone winter one-year mean model** (*colour morph ~ 1yr mean winter temperature + 1yr mean winter precipitation*) (Supplementary table 2, Mediterranean zone winter).


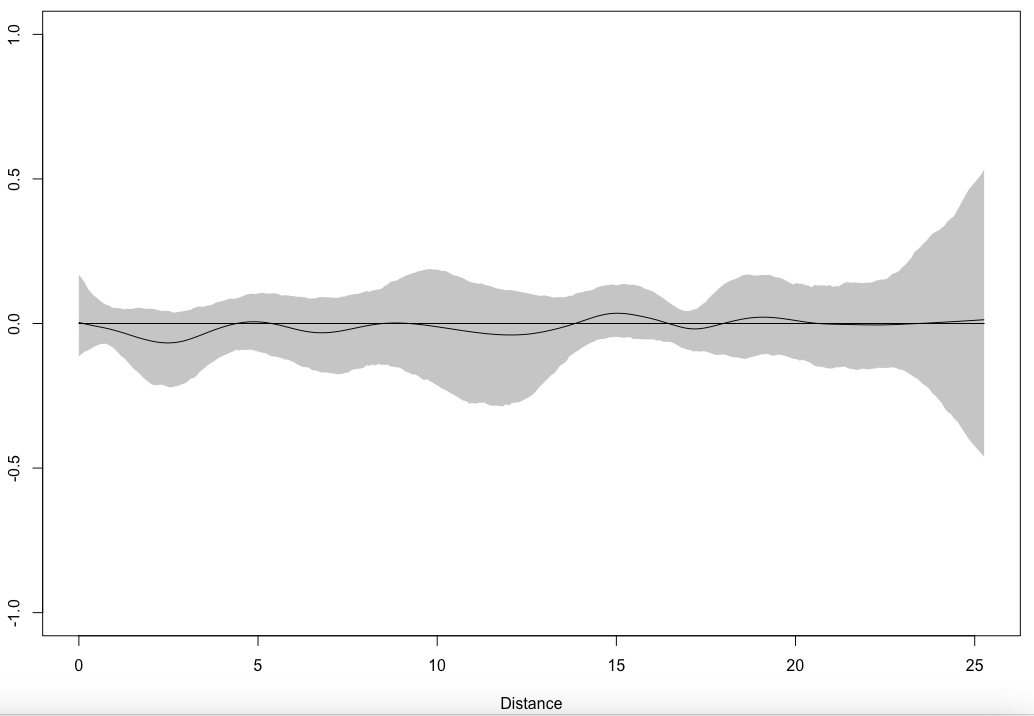


Supplementary figure 14. Spatial autocorrelation correlogram based on residuals of **Mediterranean zone summer one-year mean model** (*colour morph ~ 1yr mean summer temperature + 1yr mean summer precipitation*) (Supplementary table 2, Mediterranean zone summer).
